# Altered dynamic intrinsic brain activity of the default mode network in Alzheimer’s disease: A resting-state fMRI study

**DOI:** 10.3389/fnhum.2022.951114

**Published:** 2022-08-17

**Authors:** Zhengluan Liao, Wangdi Sun, Xiaozheng Liu, Zhongwei Guo, Dewang Mao, Enyan Yu, Yan Chen

**Affiliations:** ^1^Department of Clinical Medicine, Medical College of Soochow University, Suzhou, China; ^2^Department of Geriatric VIP No. 3 (Department of Clinical Psychology), Rehabilitation Medicine Center, Zhejiang Provincial People’s Hospital, Affiliated People’s Hospital, Hangzhou Medical College, Hangzhou, China; ^3^Second Clinical Medical College, Zhejiang Chinese Medical University, Hangzhou, China; ^4^The Second Affiliated Hospital and Yuying Children’s Hospital, Wenzhou Medical University, Wenzhou, China; ^5^Tongde Hospital of Zhejiang Province, Hangzhou, China; ^6^Department of Radiology, Zhejiang Provincial People’s Hospital, Affiliated People’s Hospital of Hangzhou Medical College, Hangzhou, China; ^7^Department of Psychiatry, Cancer Hospital of the University of Chinese Academy of Sciences (Zhejiang Cancer Hospital), Institute of Basic Medicine and Cancer (IBMC), Chinese Academy of Sciences, Hangzhou, China

**Keywords:** Alzheimer’s disease, mild cognitive impairment, resting-state functional magnetic resonance imaging, regional homogeneity, default mode network

## Abstract

**Objective:**

Static regional homogeneity (ReHo) based on the resting-state functional magnetic resonance imaging (rs-fMRI) has been used to study intrinsic brain activity (IBA) in Alzheimer’s disease (AD). However, few studies have examined dynamic ReHo (dReHo) in AD. In this study, we used rs-fMRI and dReHo to investigate the alterations in dynamic IBA in patients with AD to uncover dynamic imaging markers of AD.

**Method:**

In total, 111 patients with AD, 29 patients with mild cognitive impairment (MCI), and 73 healthy controls (HCs) were recruited for this study ultimately. After the rs-fMRI scan, we calculated the dReHo values using the sliding window method. ANOVA and *post hoc* two-sample *t*-tests were used to detect the differences among the three groups. We used the mini-mental state examination (MMSE) and Montreal Cognitive Assessment (MoCA) to evaluate the cognitive function of the subjects. The associations between the MMSE score, MoCA score, and dReHo were assessed by the Pearson correlation analysis.

**Results:**

Significant dReHo variability in the right middle frontal gyrus (MFG) and right posterior cingulate gyrus (PCG) was detected in the three groups through ANOVA. In *post hoc* analysis, the AD group exhibited significantly greater dReHo variability in the right MFG than the MCI group. Compared with the HC group, the AD group exhibited significantly increased dReHo variability in the right PCG. Furthermore, dReHo variability in the right PCG was significantly negatively correlated with the MMSE and MoCA scores of patients with AD.

**Conclusion:**

Disrupted dynamic IBA in the DMN might be an important characteristic of AD and could be a potential biomarker for the diagnosis or prognosis of AD.

## Introduction

Alzheimer’s disease (AD) is a degenerative disease mainly characterized by the deposition of β-amyloid (Aβ) protein (Gouras et al., 2015), senile plaques (Han et al., 2019), and neurofibrillary tangles (Gallardo and Holtzman, 2019), and accounts for approximately 60% of the cases of dementia. A large number of studies have shown that early detection of and early intervention for AD can improve the prognosis of patients with the disease. Mild cognitive impairment (MCI) is considered a predementia clinical condition (Geslani et al., 2005), and early recognition and treatment can delay progression to dementia. MCI is typically identified based on the observations of cognitive decline in an individual or their caregivers’ complaints and scores from a cognitive screening scale. As a result, the diagnosis of MCI and its differentiation from AD are affected by subjective factors and the patients’ education level. Therefore, objective MCI biomarkers are needed to more easily and reliably identify patients with MCI. However, there is still a lack of sensitive biomarkers for early diagnosis, while existing methods, such as cerebrospinal fluid or plasma phosphorylated Tau protein (*p*-Tau), are invasive and lack consistent standards, making them difficult to be widely carried out in the clinical practice. Consequently, a non-invasive but highly sensitive and universally applicable early diagnostic indicator is needed urgently.

Developments in the rapid magnetic resonance imaging (MRI) technology have resulted in the widespread use of resting-state functional MRI (rs-fMRI) in the study of brain mechanisms in AD and MCI because of its non-invasiveness, convenience of use, and reproducibility (Ibrahim et al., 2021). Previous studies have shown that abnormal functioning of the default mode network (DMN) according to rs-fMRI is a major pathological feature of patients with AD (Koch et al., 2012). The precuneus (PCu) and posterior cingulate gyrus (PCG) are the core of the DMN. Independent component analysis (ICA) methods have shown that patients with AD have lower functional connectivity (FC) in the DMN (specifically the PCu and PCG) than MCI and healthy controls (HCs) (Binnewijzend et al., 2012). He et al. (2007) found that the regional homogeneity (ReHo) index of the PCG/PCu significantly decreased with the progression of this disease as measured using cognition scores.

However, the aforementioned studies are based on the assumption that the blood oxygen-dependent signal remains stable during the entire MRI scanning process, ignoring dynamic changes in intrinsic brain activity (IBA) over time; therefore, they do not better reflect the pathological mechanism of AD. We hope to find a more sensitive rs-fMRI marker to evaluate AD and MCI. To date, few studies have focused on the dynamic IBA and substantial fluctuations in cognitive dysfunction. Recent studies have reported that the regional dynamic characteristics of brain activity can be captured more effectively using the dynamic sliding window method throughout the scanning procedure (Zuo et al., 2013). Compared with the HCs, patients with AD exhibited high-dynamic amplitude of low-frequency fluctuations (dALFFs) variability in the bilateral cerebellar posterior lobe and the left middle temporal gyrus (Li et al., 2021). The dALFF values of the right parahippocampal gyrus in patients with MCI also show a significant interaction effect with the fluorodeoxyglucose hypometabolic aggregation index (Lin et al., 2021).

By dividing the whole time series into a series of windows, dynamic ReHo (dReHo) technology can be used to calculate the time variability of the ReHo index (Sun et al., 2021), which can reflect the dynamic activity mode of the region (Zuo et al., 2013). In this way, dReHo, unlike static ReHo, may better reflect the time-dependent dynamic characteristics of IBA and describe the dynamic properties of regional temporal synchronization of IBA between adjacent voxels, reflecting fluctuations in IBA (Deng et al., 2016). Deng et al. (2016) found that the brain region with the highest dReHo variability tended to be the center of brain activity and was related to the reorganization of brain function. Therefore, we have reason to believe that dReHo can be used as a sensitive indicator for exploring changes in IBA in AD. Nevertheless, studies of dReHo in patients with AD are lacking, and the mechanism of dynamic local consistency in the DMN of patients with AD remains unclear.

In this study, we obtained rs-fMRI data to compare the differences in dReHo among the AD, MCI and HC groups. The relationships between dReHo variability and neuropsychological scales in MCI and AD were also explored. Based on the previous studies, we hypothesized that AD would show abnormal dReHo values in the DMN compared with MCI and HC and that alterations would be associated with the severity of the disease.

## Materials and methods

### Patients and clinical assessment

All the patients with AD and MCI were recruited from the Department of Clinical Psychology in the Zhejiang Provincial People’s Hospital, and the HCs were recruited from the community between January 2017 and December 2019. A total of 168 patients with AD, 31 patients with MCI, and 74 HCs were enrolled. In total, sixty subjects were excluded because of the clinical data loss (40 patients with AD and 1 for patient with MCI) and head movement exceeding the standard limits during the MRI scan (17 patients with AD, 1 patient with MCI, and 1 HC). All the participants were between 55 and 85 years of age, could be of any sex, were right-handed, and were capable of completing all the neuropsychological assessments and MRI scans. All the participants were first-time patients and had not previously taken any cognitive-promoting medication. Finally, 111 patients with AD, 29 patients with MCI, and 73 HCs were enrolled. This study complies with the Declaration of Helsinki and was approved by the Ethics Committee of Zhejiang Provincial People’s Hospital. All the subjects or their family members signed informed consent forms.

All the participants in this study were recruited in accordance with our previous fMRI-based AD and MCI temporal function connection study (Li et al., 2021). The details of the inclusion and exclusion criteria, data collection and processing, and quality control were all obtained in the previous studies (Li et al., 2021).

The patients with AD conformed to the National Institute of Neurological and Communicative Disorders and Stroke and the AD and Related Disorders Association (NINCDS–ADRDA) criteria for “probable AD” (McKhann et al., 2011). Patients with MCI met the Petersen’s criteria (Petersen et al., 2001), had a Clinical Dementia Rating (CDR) score of 0.5, complained of memory loss while otherwise demonstrating well-preserved cognitive functions, and indicated a degree of independence in daily life exceeding that seen in dementia. The HC group had no neurological or psychiatric disorders and no cognitive impairment with CDR score of 0 and mini-mental state examination (MMSE) > 26.

Neuropsychological assessment of the participants was performed using the MMSE and Montreal Cognitive Assessment (MoCA) to evaluate cognitive functions. Subjects were evaluated on a one-to-one scale by uniformly trained examiners in a quiet standardized psychological assessment room.

### Data acquisition

All the image scans were performed on a 3.0 T MRI scanner (3.0 T Discovery MR750, GE Healthcare) at the Zhejiang Provincial People’s Hospital with an 8-channel-phased array head coil to limit head movement and earplugs to reduce the impact of noise. The images of the resting state function were collected by an echo-planar imaging (EPI) sequence, and the parameters were as follows: TR = 2,000 ms; TE = 30 ms; field of view (FOV) = 220 × 220 mm; flip angle = 78°; slice thickness = 3.4 mm; voxel size: 3.4 × 3.4 × 3.2 mm^3^; forty-four axial slices and 200 volumes covering the entire brain were obtained in 6 min and 40 s. In addition, T1 images were refocused using a spoiled gradient refocused acquisition sequence (TR = 2,300 ms; TE = 2.26 ms; turning angle = 8°; slice thickness = 1 mm; FOV = 256 × 256 mm; voxel size = 1 × 1 × 1 mm^3^).

### Data preprocessing

Data Processing Assistant for Resting-State fMRI (DPARSF) version 4.0^1^ and SPM8^2^ were used to preanalyze the functional images acquired in our study. The preprocessing of each subject was performed as follows: (1) removal of the first 10 time points for steady-state magnetization and participant adaptation; (2) slice timing correction; (3) realignment: participants whose head motion exceeded 3.0 mm of maximum displacement or 3.0° of rotation were excluded; (4) spatial normalization to the Montreal Neurological Institute (MNI) space *via* the deformation fields derived from the tissue segmentation of structural images (resampling voxel size = 3 × 3 × 3 mm^3^); and (5) nuisance covariate regression (Friston’s 24 head-motion parameters, white matter signal and cerebrospinal fluid [CSF] signal). (6) Temporal filtering was conducted in a typical band (0.01–0.08 Hz).

### Computation of dynamic regional homogeneity

The dReHo was computed using sliding-window analysis by the temporal dynamic analysis (TDA) toolkits based on DPABI (Yan et al., 2017). First, the size of the moderate-length sliding window was set to 30 TR to capture brain dynamics, and the step size of its movement was set at 1TR. Second, the ReHo map for each sliding window was computed to evaluate dReHo for all the windows. Third, to assess the temporal variability in brain activity, the coefficient of variation (CV) of the dReHo maps across windows was calculated. At last, all the CV maps were smoothed with a Gaussian kernel of 6 mm full-width at half-maximum (FWHM).

### Statistical processes

Sex differences were analyzed by the *χ^2^* test. One-way ANOVA and *post hoc* analysis were used to assess differences in age, duration of education, MMSE, and MoCA scores between patients with AD and MCI and HCs using SPSS (version 24.0, Armonk, NY, United States) with a threshold of *P* < 0.05. We applied ANOVA and *post hoc* two-sample *t*-tests to assess whether dReHo was significantly different between the groups. The head motion parameters (mean root mean square values), age, and sex were regressed as covariates. The resultant *t*-maps were corrected for multiple comparisons using AlphaSim, a Monte Carlo clusterwise simulation program implemented in AFNI^3^. The statistical threshold was set at *P* < 0.001 with a cluster size > 13 voxels, which corresponded to a corrected *P* < 0.05. All the coordinates are displayed in the MNI coordinates, as used by SPM.

### Relationship of dynamic regional homogeneity with clinical variables

In addition, an analysis was performed to identify the association between the neuropsychological scale (MMSE and MoCA) scores and dReHo in the AD and MCI groups. The mean dReHo signal of the abnormal brain regions was extracted for the Pearson’s correlation analysis. Correlation analysis was performed with Graph Pad Prism 6 at a significance threshold of *P* < 0.05 (uncorrected).

## Results

### Demographics and clinical data

A total of 111 patients with AD, 29 patients with MCI, and 73 HCs were finally included in the data analysis. No significant differences in age (*F* = 1.27, *P* = 0.28), sex (χ^2^ = 2.62, *P* = 0.27), or education level (*F* = 0.33, *P* = 0.72) were found among AD, MCI, and HC groups (Table 1). The MMSE (*F* = 186.81, *P* < 0.001) and MoCA (*F* = 179.90, *P* < 0.001) scores were significantly different among the three groups (Table 1). In addition, *post hoc* analysis indicated that the significant differences in MMSE and MoCA scores were present between each group (*P* < 0.001).

**TABLE 1:** Demographic and clinical information.

	AD (*n* = 111)	MCI (*n* = 29)	HC (*n* = 73)	χ^2^/F	*P*
Gender (male)	37	13	32	2.62	0.27
Age (mean ± SD)	68.28 ± 9.64	65.90 ± 10.05	66.30 ± 9.52	1.27	0.28
Education (mean ± SD)	7.87 ± 4.41	8.45 ± 4.60	8.26 ± 3.38	0.33	0.72
MMSE score (mean ± SD)	17.24 ± 5.57	25.97 ± 0.94	28.77 ± 0.83	186.81	<0.001*
MoCA score (mean ± SD)	13.39 ± 6.30	21.21 ± 3.83	27.19 ± 1.66	179.90	<0.001*

The χ^2^ test was performed to assess differences in gender between groups. One-way ANOVA with the Bonferroni post hoc test was performed to assess differences in age, education level, MMSE, and MoCA scores; the mean values are expressed as x ± s.

*P < 0.05 for the significant differences among groups.

HC, healthy control; MCI, mild cognitive impairment; AD, Alzheimer’s disease; MMSE, mini-mental state examination; MoCA, Montreal Cognitive Assessment.

### Differences in dynamic regional homogeneity analysis

**FIGURE 1:**
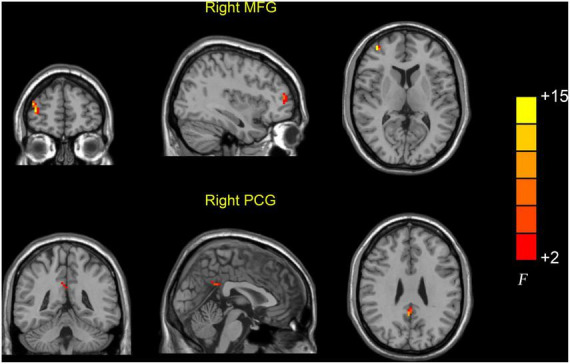
Brain regions showing abnormal dReHo values among the AD, MCI, and HC groups. Differences in dReHo were shown in the right MFG and PCG among the three groups using ANOVA. HC, healthy control; MCI, mild cognitive impairment; AD, Alzheimer’s disease; MFG, middle frontal gyrus; PCG, posterior cingulate gyrus.

**FIGURE 2:**
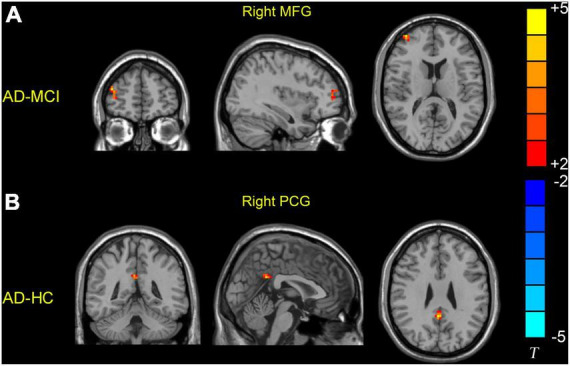
Brain regions showing increased dReHo values in the AD group relative to the MCI and HC groups. **(A)** Patients with AD showed higher dReHo values in the right MFG than patients with MCI. **(B)** Patients with AD showed higher dReHo values in the right PCG than the HC group. HC, healthy control; MCI, mild cognitive impairment; AD, Alzheimer’s disease; MFG, middle frontal gyrus; PCG, posterior cingulate gyrus.

Significant dReHo variability was identified in the right middle frontal gyrus (MFG) (peak MNI: 39, 54, and 9) and right PCG (peak MNI: 3, −51, and 30) in the three groups through ANOVA (Table 2 and Figure 1). *Post hoc* two-sample *t*-test results showed that the AD group exhibited significantly greater dReHo variability in the right MFG (peak MNI: 39, 57, 15) than the MCI group (Table 2 and Figure 2A). Compared with the HC group, the AD group exhibited significantly increased dReHo variability in the right PCG (peak MNI: 3, −48, and 30) (Table 2 and Figure 2B). However, there was no significant difference in dReHo values between the MCI and HC groups.

**TABLE 2:** Brain regions with significant differences in dReHo among groups.

Brain regions	Cluster size (voxels)	BA	Peak MNI coordinates			Peak F/*t* values
			
			X	Y	Z	
**ANOVA**						
Right middle frontal gyrus	28	46	39	54	9	12.35
Right posterior cingulate gyrus	15	23	3	−51	30	11.82
**AD** vs. **MCI**						
Right middle frontal gyrus	25	46	39	57	15	4.41
**AD** vs. **HC**						
Right posterior cingulate gyrus	26	23	3	−48	30	4.56

dReHo, dynamic regional homogeneity; BA, Brodmann area; MNI, Montreal Neurological Institute; ANOVA, analysis of variance; HC, healthy control; MCI, mild cognitive impairment; AD, Alzheimer’s disease.

### Correlations with clinical features

**FIGURE 3:**
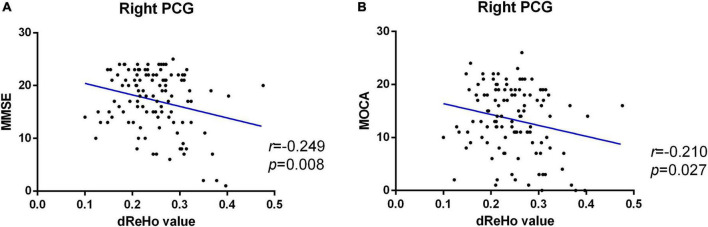
Significant negative correlation between altered dReHo variability in the right PCG and MMSE **(A)** and MoCA **(B)** scores of patients with AD. dReHo, dynamic regional homogeneity; AD, Alzheimer’s disease; PCG, posterior cingulate gyrus; MMSE, mini-mental state examination; MoCA, Montreal Cognitive Assessment.

Pearson correlation analysis was used to calculate the correlation between the dReHo value and neuropsychological scores and showed that in patients with AD, the dReHo variability in the right PCG was significantly negatively correlated with MMSE (*r* = −0.249, *P* = 0.008) (Figure 3A) and MoCA (*r* = −0.210, *P* = 0.027) (Figure 3B) scores.

## Discussion

To our knowledge, this is the first attempt to apply dReHo to investigate dynamic IBA in a large sample of individuals including patients with AD and MCI and HCs, addressing the limitation of most of such studies involving only small sample sizes and two groups for comparison. In the current study, we investigated the alterations of dynamic IBA in patients with AD, MCI, and HCs using resting-state dReHo analysis. First, we showed that the right MFG and right PCG demonstrated different levels of dReHo variability in the AD, MCI, and HC groups. Compared with the HCs group, the AD group exhibited significantly increased dReHo variability in the right PCG. The AD group also displayed significantly greater dReHo variability in the right MFG than the MCI group. Both of these aforementioned regions are mainly involved in the DMN. In addition, the dReHo variability in the right PCG was significantly negatively correlated with the MMSE and MoCA scores in patients with AD.

The dReHo variability in the right PCG of patients with AD was significantly greater than that of HCs, which indicates that the fluctuation in IBA in the right PCG is unstable in patients with AD. The PCG is located in the posterior DMN; this brain region is not only the core brain region of the DMN but also the most active brain region involved in IBA. Previous studies have shown that the PCG is widely involved in episodic memory, working memory, and short-term memory processing (Leech and Sharp, 2014), indicating a role in the progression of AD. A number of studies have indicated that static IBA in the PCG is disrupted in patients with AD. Zhang et al. (2021) found that static ALFF and ReHo are decreased in the PCG in patients with AD. Cha et al. (2015) also found that FC in the PCG of patients with AD was lower than that of normal elderly people. Our study findings are consistent with the above results and expand the characteristics of the destabilization of IBA in the PCG of patients with AD from the perspective of dynamic local synchronization, greatly improving our understanding of the pathological process affecting the PCG in AD. In addition, we found that dReHo of the right PCG was negatively correlated with the MMSE and MOCA scores, suggesting that the fluctuation in dynamic IBA in the right PCG is positively correlated with the severity of AD and that it could be used to predict disease progression, which is consistent with the findings from static studies (Leech and Sharp, 2014; Lee et al., 2020). Therefore, the combination of the two can better reflect the disease severity of AD. Buckner et al. (2009) believed that the PCG is the most important center of intrinsic connectivity of the cerebral cortex, which has high-neuronal activity. However, the higher intrinsic neuronal activity predisposes patients to an increase in Aβ production, leading to its deposition in this brain region (Cirrito et al., 2005). Therefore, we speculate that the PCG may be the earliest brain region that experiences Aβ deposition, contributing to the instability of dynamic IBA, resulting in AD-related cognitive impairment symptoms and participating in the pathological process of AD. This study further confirmed the important role of the PCG in the internal mechanism of AD from the perspective of dynamic IBA.

In addition, greater dReHo variability in the right MFG was detected in patients with AD than in patients with MCI, which demonstrated that dynamic IBA of the right MFG in patients with AD is more unstable than that in patients with MCI. The MFG is a non-negligible brain region of the anterior DMN, which is involved in IBAs such as attention maintenance (Song et al., 2019), dealing with the working memory (Rajah et al., 2011) and language processing (Koyama et al., 2017). Previous studies have found that static IBA of the MFG is disrupted in patients with AD. Weiler et al. (2014) found that static ALFF is decreased in the MFG in patients with AD. Studies have also found that the static ReHo value is decreased in the MFG in both patients with MCI and AD (He et al., 2007; Long et al., 2016). Another study found that the FC within the anterior DMN was enhanced in the early stage of AD but decreased with disease progression (Zhao et al., 2020), similar to the results of our study. Our findings supplement the dynamic local synchronization changes in the MFG observed in patients with AD and MCI. Hence, we conclude that the dynamic IBA in MFG becomes more unstable with disease progression, which may help identify individuals from MCI to AD from the perspective of dynamic IBA and understanding these changes throughout the transition may help delay the transition from MCI to AD.

We did not find dynamic IBA abnormalities between MCI and HC, which could be because of a number of factors. For example, the MCI may still be in the early stage, and the presence of subthreshold Aβ deposition alone is not sufficient to produce functional imaging changes (Qi et al., 2010; Esposito et al., 2013). Static ReHo studies have found that patients with MCI have higher ReHo values in some brain regions than HCs (Cai et al., 2018; Hu et al., 2021), suggesting a possible compensatory mechanism.

Taken together, these results indicate that AD displays a disrupted dynamic IBA in the DMN, which provides a new perspective for uncovering the underlying neuropathological mechanism of AD. The dReHo variability of the PCG and MFG might be an important characteristic change in AD and MCI and thus could be a potential biomarker for the diagnosis or prognosis of AD.

In addition, sliding window length and step size are the main influences on the results of dynamic fMRI (Hutchison et al., 2013; Li et al., 2020). We established two combinations to verify the stability of the results of this study: (1) different sliding window lengths (40s, 80s) and step size (2s), (2) sliding window length 60 and step size 4s. The results for different window widths and step sizes show differential brain regions are consistent. Meanwhile, the DMN is the brain network with relatively high consistency of results in dynamic fMRI analysis (Li et al., 2020).

The limitations of this study cannot be ignored. First, although patients were recruited from the three phases of the disease, it remains a cross-sectional study. Longitudinal studies are still necessary to investigate the dReHo changes during disease progression and to determine the pathology of dynamic IBA changes. Second, we considered only the total MMSE and MoCA scores, not the subitem scores. A more complete scale (such as the delayed recall test of Webster’s Memory Scale–Logical Memory II and the immediate and delayed memory parts of the Auditory Verbal Learning Test) would be helpful to explore the relationship with dReHo and each cognitive function.

## Conclusion

We investigated the alterations in dynamic IBA in patients with AD and MCI and HCs using resting-state dReHo analysis. The results indicated that AD displays a disrupted dynamic IBA in the DMN, which provides a new view for revealing the underlying neuropathological mechanism of AD. The dReHo variability of the PCG and MFG might be an important characteristic change in AD and could be a potential biomarker for the diagnosis or prognosis of AD.

## Data availability statement

The original contributions presented in this study are included in the article/supplementary material, further inquiries can be directed to the corresponding authors.

## Ethics statement

The studies involving human participants were reviewed and approved by the Zhejiang Provincial People’s Hospital. The patients/participants provided their written informed consent to participate in this study. Written informed consent was obtained from the individual(s) for the publication of any potentially identifiable images or data included in this article.

## Author contributions

YC, EY, and ZL designed the experiments. YC and WS collected and sorted the data. XL and DM carried out the experiments, data analysis, and statistical analysis. XL and ZG assisted with the statistical analysis. YC and ZL wrote the manuscript. YC, ZL, XL, and ZG revised the manuscript. All authors contributed to the article and approved the submitted version.

## Footnotes

1
http://www.rfmri.org

2
https://www.fil.ion.ucl.ac.uk/spm/

3
http://afni.nimh.nih.gov
